# Cognitive Planning Improved After Cycling Exercise in Older Adults with Down Syndrome

**DOI:** 10.3390/brainsci15010002

**Published:** 2024-12-24

**Authors:** Shannon D. R. Ringenbach, Nathaniel E. Arnold, Forouzan Rafiei Rezvani, Chih-Chia Chen

**Affiliations:** 1 College of Health Solutions, Arizona State University, Phoenix, AZ 85004, USA; nearnol1@asu.edu (N.E.A.); frafieir@asu.edu (F.R.R.); 2 Department of Health and Human Physiology, University of Iowa, Iowa City, IA 52242, USA; chih-chia-chen@uiowa.edu

**Keywords:** executive function, physical activity, intellectual deficit, trisomy 21, intervention, adults

## Abstract

Background/Objectives: Cognitive functions are a crucial part of daily living, especially for adults with Down syndrome (DS) who have a high likelihood of developing Alzheimer’s disease in adulthood. In addition, adults with DS move slower and are not meeting the standard aerobic activity guidelines each week. The aim of this study was to examine if Assisted Cycle Therapy (ACT) would improve cognitive planning as measured by the Tower of London (TOL), set switching as measured by the modified Wisconsin Card Sorting Test, and spatial memory as measured by the Corsi Block Test in adults with DS as compared to self-paced cycling. Methods: Twenty-four participants were randomly assigned to one of two interventions over eight weeks. (1) Thirteen older adults with DS completed the ACT intervention, which is stationary cycling with the assistance of a motor to maintain a cadence at least 35% greater than voluntary cycling. (2) Eleven older adults with DS completed voluntary cycling (VC). Results: Our results showed that cognitive planning as measured by total correct score in the TOL showed improvement for both ACT and VC after 8 weeks of exercise, F(1, 22) = 6.22, *p* = 0.021. There were no significant differences for spatial memory or set switching. Conclusions: We concluded that cycling exercise has a positive impact on cognitive function, especially problem solving in older adults with DS. Our results are discussed with respect to upregulation of neurotrophic factors that increase functioning in the prefrontal cortex that accompanies exercise and leads to improvements in cognitive planning which is essential to many activities of daily living and quality of life for older adults with DS.

## 1. Introduction

Down syndrome (DS) is a genetic disorder caused by an extra 21st chromosome. The most distinguishing characteristic in people with DS is a deficit in cognitive function. Executive functions encompass an extensive set of higher-order cognitive operations that organize and regulate goal-orientated tasks within the prefrontal cortex [1]. Executive functions is a term that includes cognitive skills such as planning, memory, attention, and set switching, which are essential for activities of daily living. Specifically, it was recently reported that cognitive planning was related to comprehension of narrative and expository text in first graders [2]. Over 20 years of research in my lab has shown that generally, adults with DS have an IQ similar to a typical first grader, which makes this finding relevant. Furthermore, Alzheimer’s disease (AD), which is a serious dysfunction of global cognitive control occurs three to five times greater in adults with DS and at a much earlier age than the general population [3]. Today people with DS are living into their 60’s, thus it is vital to improve and/or maintain cognitive planning and throughout the lifespan to continue their quality of life.

Another contributing factor to cognitive function in people with DS is that they prefer to lead a sedentary lifestyle. A recent study investigating lifestyle physical activity measured with actigraph monitors and its effect on behavioral and neural imaging measures in adults with DS reported that in general, greater time in sedentary behavior was associated with worse cognitive performance and more dementia symptoms using behavioral measures [4]. Alternatively, moderate-to-vigorous activity was positively associated with cognitive functioning and fewer dementia symptoms, although there were no changes to amyloid-beta and tau [4]. Furthermore, these authors suggested a need for longitudinal studies to determine if physical activity promotes cognitive health in adults with DS, which is the goal of the present study. The present study also uses behavioral measures of cognitive function, which have been shown to be sensitive to physical activity in persons with DS.

Diamond [5] demonstrated neurobehavioral evidence that the cerebellum, basal ganglia, prefrontal cortex, and neurotransmitters (e.g., dopamine) are involved in both motor and cognitive development into adulthood. A recent review showed positive relationships between fine and gross motor skills and reading in typical children and adolescents [6]. Another study showed a small, positive relationship among children revealed a general cognitive and motor performance relationship among typical and atypical children (5–6 yrs.) [7]. One study in adolescents with DS (Mchronological age = 20.6 yrs., MMental age = 5.75 yrs) measured executive function prior to and after a single bout of 20 min of moderate intensity treadmill walking exercise [8]. Following this acute exercise intervention inhibition improved and it was suggested that increased brain-derived neurotrophic factor may be an explanation. Previous research in our lab has found that 8 weeks of 3 times/week for 30 min of Assisted Cycling Therapy (ACT), which is cycling with a motor that moves legs at least 35% faster than they can move on their own, can promote cognitive improvements in young adults (Mchronological age = 19.5 yrs., Mmental age = 5.6 yrs.) with DS [9]. ACT exercise improved reaction times and inhibitory control over voluntary rate cycling and both cycling exercises improved semantic language fluency. These results were interpreted to suggest that the motor activity created neuroplasticity in the prefrontal cortex, which lead to improvements in cognitive function that could indicate changes at the neuronal level.

To our knowledge, there are no studies investigating exercise interventions on cognitive function in older adults with DS. This is important because people with DS are living longer and have an increased risk of Alzheimer’s disease. Thus, interventions to improve their cognitive function is crucial to maintaining their quality of life. Based on our previous research with adolescents with DS [9] and the influence of ACT on cognitive measures of executive function, we predicted that adults with DS would improve in cognitive planning as measured by the TOL, set switching as measured by the modified Wisconsin Card Sort, and spatial memory as measured by the Corsi Block Test after 8 weeks of ACT and voluntary cycling. Furthermore, we predicted that ACT would improve cognitive function more than VC, based on previous research that showed that ACT may produce more neurological changes and cognitive benefits.

## 2. Materials and Methods

### 2.1. Participants

Participants consisted of 24 adults aged 26 yrs. and older with Down syndrome, with a mean age of 37.12 yrs. and a mean mental age of 6.6 yrs. Participants were recruited through flyers, word of mouth in the community, phone calls, and email announcements. Parents or legal guardians completed a Physical Activity Readiness Questionnaire on behalf of the participant to determine the participant’s eligibility and readiness for exercise. The inclusion criteria included (1) answering ‘No’ to all seven questions of the Physical Activity Readiness Questionnaire (PARQ), or having received exercise clearance from their physician using the Physical Activity Readiness Medical Exam, (2) report of trisomy 21 type of DS, (3) report of age older than 26, (4) report of no physical disabilities that would limit cycling exercise. The exclusion criteria included (1) cardiopulmonary disease or stroke, and (2) current participation in a formal exercise intervention.

All participants and their legal guardians completed informed assent and/or consent forms. All protocols used in this intervention are approved by the Human Subjects Institutional Review Board of Arizona State University.

Thirteen participants were placed in the Assisted Cycle Therapy (ACT) group and eleven were placed in the voluntary cycling (VC) group.

### 2.2. Design

Participants were randomly assigned to two groups during their 8-week intervention. The groups included ACT (*n* = 13) and VC (*n* = 11). Baseline tests were administered a week prior to their 8-week intervention and post-testing was completed within 1 week of completion of the intervention.

### 2.3. Intervention

The use of a bike with a motor was used to implement the therapy of assisted cycling or voluntary cycling. Prior to the using the bike for each session, the calibration of the SRM power control was completed. Next, a heart rate monitor was placed on participants, which was monitored throughout the cycling session. During the beginning of each exercise intervention, the participants were seated for five minutes, and resting heart rate was recorded at the end of the rest period. The seat height and the distance of the bicycle from pedals was adjusted for each individual and recorded for future reference for all following testing sessions. Sufficient familiarization/practice time on the bicycle was afforded to the participants, as many of the participants had never ridden a bicycle before the intervention. Heart rate was allowed to return within 5% of resting level in a short break after the practice period. Based on the intervention randomly assigned to the participant at the beginning of the study, the 30-min cycling session was completed. During each cycling session, the RPE scale was implemented to engage and record the perception of difficulty while participants biked. The scores ranged on a scale of 1 to 4; 1 being “easy”, 2 “kind of easy”, 3 “hard”, and 4 was “very hard”. These were asked of each participant during their cycling session and at each stage at the 5-min mark, along with their heart rate and cadence based on the SRM power control device.

#### 2.3.1. Assisted Cycling Therapy (ACT)

Assisted Cycling Therapy participants pedaled at a pre-determined rate with the help of a mechanical motor that moved their legs. The motor was programmed to maintain a cadence which was at least 35% faster than the voluntary cadence for 30 min.

#### 2.3.2. Voluntary Cycling (VC)

Voluntary cycling occurred on the same stationary bicycle but did not utilize the mechanical motor, allowing the participants to pedal at their own self-selected rate for 30 min of active exercise.

### 2.4. Descriptive Measures

The Tower of London (TOL) has been shown to have good validity evaluating problem solving in adults with Down syndrome and has been highly related to other measures of executive functioning [10]. This test utilized a wooden platform with a total of three pegs with graduating heights and three wooden balls (painted blue, red, and yellow). The goal of each trial was to reproduce a certain arrangement of the balls depicted on a picture by moving only one ball at a time. The rules for each trial consisted of replicating the image in a certain number of moves and within a certain time limit (i.e., 30–60 s). Participants were told to move only one ball at a time and that each move must end with the ball on one of the three pegs, which still had available space. The rules excluded placing more than one ball on a peg that had no more space or taking two places at once or the placement of one ball on the table. After one practice trial, participants moved through 17 different trials of increasing complexity and difficulty. Each trial was considered a success if the participant achieved the goal of arranging the platform in the given time limit, within the number of moves, and if the pattern was accurate. The test ended when the participant completed all the trails or when the participant failed to complete 4 consecutive trials, either due to time limitations, failure in following the rules, completing the arrangement in an excessive number of moves, or if the pattern was not correct. For the TOL three measures were used. The total correct score is the number of items completed with the correct pattern, in the correct number of moves, within the given time limit. The total correct moves is the sum of moves taken in completed levels. The total aggregate score is the average time per correct move/total number of correct moves.

An extensive literature review on visuo-spatial abilities in persons with DS was conducted and stated that the Corsi Block task is by far the most commonly used visuo-spatial task in the DS literature [11]. The Corsi Block test was utilized to evaluate a participant’s visuo-spatial short-term memory. The test took place on a laptop that was placed in the center in front of the participant, who was instructed to navigate the mouse in order to click on the squares. If a participant was not familiar with the use of a mouse, then they were instructed to point at the squares in the screen and the researcher administering the test would click on the corresponding squares. The rules explained prior to the test included watching the computer screen as a various numbers of squares changed their color from blue to yellow for 1000 ms. The squares would change color one at a time. The number of squares highlighted per trial ranged from a total of two to nine. The participant was instructed to remember the order in which the squares changed color and then asked to select those squares in the same order. The participant was allowed two practice trials and at the conclusion of each trial, feedback regarding the correctness of the trial was given. Once the practice trials were completed, the test began. There were four measures of span, number correct, total, and memory.

The modified Wisconsin Card Sorting Test is a simplification of the test for typical children aged 4–13 yrs. which has shown validity in measuring set shifting ability and has been previously used to assess cognitive flexibility in adolescents with DS [12]. For the Card Sorting test, the participants were presented with two trays, one tray with a card of a blue rabbit (i.e., on their left-hand side) and the other tray with a card of a red boat (i.e., on their right-hand side). This test consisted of three types of trials. During the first trial, the participants were asked to sort six cards depicting either a red rabbit or a blue boat, one by one, into the corresponding tray based on its color (i.e., the red rabbit card into the tray with the card of a red boat and the blue boat card into the tray with a blue rabbit). Each card was placed face down into the corresponding tray for a total of one point per correct card placement. During the next trial, the participants were asked to sort the same set of six cards, but instead of sorting by color, participants had to sort by shape (i.e., the red rabbit card into the tray with the card of the blue rabbit and the blue boat card into the tray with the card of the red boat). Again, one point for each correct card sorted. A third trial was performed in which the participants were asked to sort the cards differently depending on if they had a border or not. In this trial, they were presented with 12 cards. If the card had a border around its edges, they had to play the color game, the participants were correct in sorting it if they sorted the card based on color (i.e., a card with a red rabbit and a border around the edges was sorted based on its red color). If the card did not have a border, they played the shape game, in which the participant was correct in sorting if they sorted the card based on its shape (i.e., a card with a red rabbit and no border around the edges was sorted based on the shape of the rabbit). In this trial, a total of 12 points were available, 1 for each correct card sorted. For all three trials, prior to beginning the test, the rules were demonstrated and described to the participant with a practice allocated at the beginning. A total of 24 points were possible for all three trials, in which the color game allocated 6 points, the shape game allocated 6 points, and the set switching test which both games utilized allocated a possible 12 points.

### 2.5. Data Analysis

Analysis of the collected data was completed using the program SPSS (24th edition). All data was analyzed using a 2 group (ACT, VC) × 2 Time (pre, post) mixed ANOVA with repeated measures on the last factor.

## 3. Results

### 3.1. Descriptive Statistics

As see in Table 1, demographic information was provided for each group (ACT, VC).

### 3.2. Cognitive Measures

**Figure 1:**
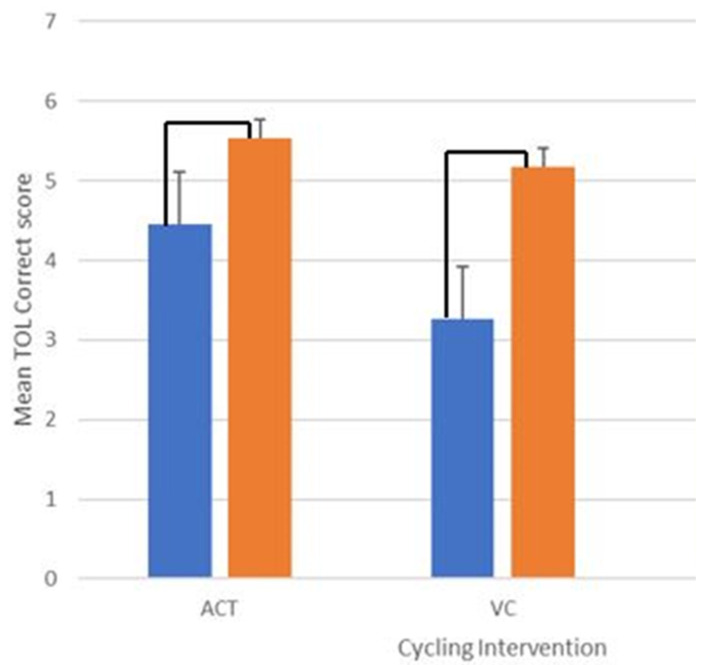
Mean correct score on the Tower of London as a function of cycling intervention (ACT, VC) and time (pre, post). Pre scores are in the first blue bar of each group and post scores are in the second orange bar of each group and the black line indicates significant differences.

As can be seen in Figure 1, for the total correct in the Tower of London, the results indicated a significant main effect for time, F(1, 22) = 6.22, *p* = 0.021, partial η^2^ = 0.220; no significant main effect for group, F(1, 22) = 24.96, *p* = 0.68, partial η^2^ = 0.008; and no significant interaction between group and time, F(1, 22) = 0.48, *p* = 0.49, partial η^2^ = 0.021.

No other significant differences were found for other measures of the TOL, Card Sorting, or Corsi Block tests.

## 4. Discussion

This is the first study, to our knowledge, which has utilized a long-term intervention (i.e., eight weeks) using Assisted Cycling Therapy (ACT) in adults with DS and that has measured changes in problem solving, set switching, and spatial memory. This was important because adults with Down syndrome are predisposed to executive function deficits, which can affect activities of daily living (e.g., reading comprehension, using public transportation, misplacing, and forgetting placement of objects, etc.).

Our results are somewhat consistent with our prediction that older adults with DS will improve in cognitive planning, set switching, and spatial memory after 8 weeks of ACT and VC following 3 times/week of 30 min of stationary cycling exercise. For cognitive planning, the total correct score in the TOL supported our prediction. In our previous research, following 8 weeks of ACT, we found improvements in cognitive planning and manual dexterity in adolescents with DS compared to no improvements following 8 weeks of voluntary cycling [13]. In another study with young adults with DS, we found improvements in other executive function tasks, such as information processing, as measured by reaction time, and inhibition as measured by the Card Sorting task following ACT, but not VC or no cycling [9].

In our single bout of exercise, we found that the measure of inhibition improved following ACT but not VC in adolescents with Down syndrome. Inhibition is an operation in executive function, which measures the ability to ignore distraction and remain focused during tasks [8]. In an 8 week intervention study from our lab also with adolescents, it was found that following 2x/week for 8 weeks of ACT, there were improved measures of processing, assessed by reaction time (RT), which is another measure of executive function. In this study there were 17 adolescents with DS that were in the Assisted Cycle Therapy group, 16 in a self-selected pace voluntary cycling group and 11 in a no cycling intervention. The results showed that the ACT group improved in reaction times and inhibitory control after 8 weeks, whereas the VC and NC groups did not improve. In addition, the VC group showed improved set-shifting following 8 weeks of cycling, whereas the ACT and NC groups did not show any improvement in set-shifting [9]. Thus, previous research with adolescents with DS aligns with the current research in older adults of improvements in executive function following ACT and VC interventions [9]. Taken together, improvements in executive function have been consistently found following ACT and VC in acute and chronic studies in adolescents and young adults with DS.

**Figure 2:**
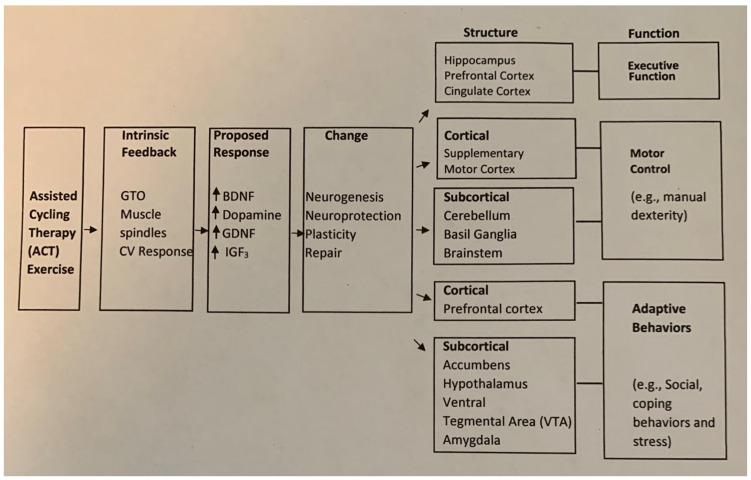
Proposed model of the mechanisms involved in ACT (modified from Alberts et al., 2011 [14]). Side errors mean that one process proceeds to the next and up errors indicate increases.

Based on Alberts’ model of forced exercise/ACT [14] and seen in Figure 2, our results have been interpreted to be due to increases in intrinsic feedback, which results in the upregulation of neurotrophic factors that produce neural change in the cortical and subcortical areas of the brain leading to improved cognitive function following exercise. Alberts and colleagues [15] have measured underlying changes in brain activity in persons with Parkinson’s disease following acute forced cycling exercise using the same bicycle that we use in our lab for ACT. fMRI data showed that the pattern of brain activation was similar after ACT and while on medication, suggesting that both interventions showed similar improvements in motor function. Other studies have found structural changes in gray and white matter volume [16] and hippocampal connectivity [17] following exercise in typical older adults and cortical silent period [18] following exercise in persons with Parkinson’s disease. Although research on how exercise impacts brain function is still in its infancy, our findings align with this interpretation. Future studies on individuals with Down syndrome (DS) should explore structural changes in cortical and subcortical regions that might explain the functional and behavioral changes observed in the current study. The results of our current study are consistent with the explanation that exercise may lead to structural changes in brain structures, leading to changes in function for older adults with DS. However, tests of executive function may be sensitive to cognitive changes by the presence or early onset of early Alzheimer’s disease in older adults with DS. This study is critical as previous research indicates that the quality of life in persons with DS is intricately linked to their physical, cognitive, and social functioning [19,20]. By understanding these relationships, this research aims to contribute to strategies that enhance the overall well-being of older adults with DS.

## 5. Limitations and Strengths

It was very difficult to recruit older participants into a true no intervention control group because guardians wanted their participant to be involved in one of the interventions. Thus, there were two groups that both exercised, which limits the likelihood of finding significant between group differences. Another limitation is that all of the measures were behavioral measures of cognitive function, which are dependent on the individual’s motivation on any particular day. Future research should include measures of brain activity and a no exercise control group for a fully randomized clinical trial design. A strength is that, because both groups exercised and were treated identically, any differences were due to the faster augmented pace in ACT versus the self-paced in VC. Another notable strength is the relatively large sample size for this population within an 8-week intervention, which yielded significant results and highlights the need for further research.

## 6. Conclusions

Teachers, therapists, and parents should be aware of the benefits of exercise and the ease of the use of a stationary bicycle at facilities where older adults with DS are known to work or spend their time. Improving executive function and all of the associated activities of daily living (e.g., reading, verbal fluency, attention, problem solving, etc.) in older adults with DS is becoming crucial as their life expectancy increases.

## Figures and Tables

**Table 1:** Descriptive Statistics.

	ACT (*n* = 13)		VC (*n* = 11)	
	Mean	SD	Mean	SD
Chronological Age (yrs.)	38.02	9.18	36.22	9.72
Mental Age (yrs.)	6.92	3.21	6.29	2.80
BMI	34.12	34.12	29.73	6.33

## Data Availability

The data presented in this study are available on request from the corresponding author due to ethical reasons.

## References

[1] Schott N., Holfelder B. (2015). Relationship between motor skill competency and executive function in children with Down’s syndrome. J. Intellect. Disabil. Res..

[2] Wu Y., Barquero L.A., Pickren S.E., Barber A.T., Cutting L.E. (2020). The relationship between cognitive skills and reading comprehension of narrative and expository texts: A longitudinal study from Grade 1 to Grade 4. Learn. Individ. Differ..

[3] Wisniewski K.E., Wisniewski H.M., Wen G.Y. (1985). Occurrence of neuropathological changes and dementia of Alzheimer’s disease in Down’s syndrome. Ann. Neurol..

[4] Fleming P.B., Patrick A., Zammit M., Alexander A., Christian B.T., Handen B., Cohen A., Klunk W., Laymon C., Ances B.M. (2021). Physical activity and cognitive and imaging biomarkers of Alzheimer’s disease in down syndrome. Neurobio Aging.

[5] Diamond A. (2000). Close interrelation of motor development and cognitive development and of the cerebellum and prefrontal cortex. Child. Dev..

[6] Macdonald K., Milne N., Orr R., Pope R. (2018). Relationships between motor proficiency and academic performance in mathematics and reading in achool-aged children and adolescents: A systematic review. Intern. J. Environ. Res. Public Health.

[7] Wassenberg R., Feron F.J., Kessels A.G., Hendriksen J.G., Kalff A.C., Kroes M., Vles J.S. (2005). Relation between cognitive and motor performance in 5-to 6-year-old children: Results from a large-scale cross-sectional study. Child. Dev..

[8] Chen C.C., Ringenbach S.D.R., Crews D., Kulinna P.H., Amazeen E.L. (2015). The association between a single bout of moderate physical activity and executive function in young adults with Down syndrome: A preliminary study. J. Intellect. Dis. Res..

[9] Ringenbach S.D.R., Holzapfel S.D., Mulvey G.M., Jimenez A., Benson A., Richter M. (2016). The effects of assisted cycling therapy (ACT) and voluntary cycling on reaction time and measures of executive function in adolescents with Down syndrome. J. Intellect. Dis. Res..

[10] García-Alba J., Esteba-Castillo S., Castellanos López M.Á., Rodríguez Hidalgo E., Ribas Vidal N., Moldenhauer Díaz F., Novell-Alsina R. (2017). Validation and Normalization of the Tower of London-Drexel University Test 2nd Edition in an Adult Population with Intellectual Disability. Span. J. Psychol..

[11] Yang Y., Conners F.A., Merrill E.C. (2014). Visuo-spatial ability in individuals with Down syndrome: Is it really a strength?. Res. Dev. Disabil..

[12] Lanfranchi S., Jerman O., Dal Pont E., Alberti A., Vianello R. (2010). Executive function in adolescents with Down Syndrome. J. Intellect. Disabil. Res..

[13] Holzapfel S.D., Ringenbach S.D.R., Mulvey G.M., Sandoval-Menendez A.M., Cook M.R., Ganger R.O., Bennett K. (2015). Improvements in manual dexterity relate to improvements in cognitive planning after Assisted Cycling Therapy (ACT) in adolescents with Down syndrome. Res. Dev. Disabil..

[14] Alberts J.L., Linder S.M., Penko A.L., Lowe M.J., Phillips M. (2011). It is not about the bike, it is about the pedaling: Forced exercise and Parkinson’s disease. Exerc. Sport. Sci. Rev..

[15] Alberts J.L., Phillips M., Lowe M.J., Frankemolle A., Thota A., Beall E.B., Feldman M., Ahmed A., Ridgel A.L. (2016). Cortical and Motor Responses to Acute Forced Exercise in Parkinson’s Disease. Park. Relat. Disord..

[16] Colcombe S.J., Erickson K.I., Scalf P.E., Kim J.S., Prakash R., McAuley E., Elavsky S., Marquez D.X., Hu L., Kramer A.F. (2006). Aerobic exercise training increases brain volume in aging humans. J. Gerontol. A Biol. Sci. Med. Sci..

[17] Burdette J.H., Laurienti P.J., Espeland M.A., Morgan A., Telesford Q., Vechlekar C.D., Hayasaka S., Jennings J.M., Katula J.A., Kraft R.A. (2010). Using network science to evaluate exercise-associated brain changes in older adults. Front. Aging Neurosci..

[18] Fisher B.E., Wu A.D., Salem G.J., Song J., Lin C.H., Yip J., Cen S., Gordon J., Jakowec M., Petzinger G. (2008). The effect of exercise training in improving motor performance and corticomotor excitability in people with early Parkinson’s disease. Arch. Phys. Med. Rehabil..

[19] Duffels M.G., Vis J.C., van Loon R.L., Nieuwkerk P.T., van Dijk A.P., Hoendermis E.S., de Bruin-Bon R.H., Bouma B.J., Bresser P., Berger R.M. (2009). Effect of bosentan on exercise capacity and quality of life in adults with pulmonary arterial hypertension associated with congenital heart disease with and without Down’s syndrome. Am. J. Cardiol..

[20] van Gameren-Oosterom H.B., Fekkes M., Buitendijk S.E., Mohangoo A.D., Bruil J., Van Wouwe J.P. (2011). Development, problem behavior, and quality of life in a population based sample of eight-year-old children with Down syndrome. PLoS ONE.

